# The Treatment of Remittent and Intermittent (Malarial) Fevers

**Published:** 1904-04

**Authors:** 


### *SELECTIONS AND ABSTRACTS.*

---

### The Treatment of Remittent and Intermittent (Malarial) Fevers.*

In this symposium by Southern authors, on "The Treatment of Remittent and Intermittent Fevers," I presume that we are expected to discuss the methods of treating malaria that are actually employed in our every-day practice. I shall therefore give simply my personal experience in treating those conditions, and will consider the etiology, pathology, and symptomatology of the different forms of malaria only in so far as is necessary to outline their rational treatment.

In discussing the prophylactic treatment of malaria, I shall confine myself to individual prophylaxis, and will not mention the various methods of destroying the malaria-bearing (Anopheles) mosquitoes. If the individual can avoid being bitten by infected mosquitoes, he of course escapes the disease; and in localities infected with malaria it is advisable for every one to sleep under mosquito bars or in carefully screened houses from early spring until winter has well begun. The patient suffering from malaria in any form should by all means be placed under a mosquito-bar as soon as the diagnosis of malaria is suspected, and kept there until he is free from every symptom, in order to avoid infecting the mosquitoes already in the home. The physician should consider it just as much a duty to instruct a family, when one of its members is infected with malaria, regarding the methods to be employed to prevent others from becoming infected, as he does when a member of a family has tuberculosis or typhoid fever. I am quite sure that the doctrine of the mosquito origin of malaria, which is becoming accepted by the laity as well as by practically all progressive physicians, has been the means of lessening the prevalence of malarial diseases all over the civilized world.

---

*By Seale Harris, M.D., Union Springs, Ala. Published in a Symposium on "The Treatment of Remittent and Intermittent Fevers" in the Therapeutic Gazette, January 15, 1904.

The value of quinine as a prophylactic in malaria is as great as when it is used for its specific effect after the disease has been contracted. From 3 to 5 grains (0.18 to 0.3 gramme) taken night and mornings will almost certainly give protection from malaria.

The Italians use as a prophylactic for malaria the fluid extract of lemon; and there is a prevalent idea among the laity in this section of the South that the juice of a lemon in a goblet of water, taken before breakfast, will prevent malaria when one is in a locality where he is exposed to the infection. If there is any virtue at all in this method it is probably due to the tonic, laxative, and cholagogic effect of the lemon juice. My observation has been that the well-nourished individual who is in perfect physical condition is but slightly susceptible to malaria, which seems to seek for its victims principally those who are weakened or prostrated from other causes; hence it is advisable for every one in a malarial locality to employ a liberal diet, and to use other means of keeping his vitality and strength up to the highest normal point. Since the person who has had malaria is peculiarly susceptible to recurrences, it is advisable to give him prophylactic doses of quinine, with iron, arsenic, and other tonics, to build him up as rapidly as possible. In some cases it is necessary for a change of climate before the patient's health is completely restored.

I am quite sure that all clinicians who have had such experience in treating intermittent and remittent fevers will agree that quinine is our safest and most reliable antimalarial remedy, but I am convinced that it is one of the most abused drugs ever in use; and were it not in the great majority of cases a harmless agent, untold damage would result from the indiscriminate and haphazard use of quinine by the laity, and even by the medical profession, in the South. There are many persons, however, who do not tolerate quinine well, and there are certain conditions, as in typhoid fever and tuberculosis, where quinine deranges the stomach, thereby interfering with nutrition, and also increases or intensifies the nervous manifestations of those diseases; so that, unless the malarial organisms have been seen in the blood, I do not think we are justified in following the plan employed by many physicians in fevers of uncertain origin—i. e., giving heroic doses of quinine for days, or even weeks, to exclude or assert the diagnosis of malaria. If the administration of quinine in antimalarial doses for forty-eight hours does not materially affect the fever in the great majority of cases, it is due to other causes than malaria; though there are occasional cases, one of which I will report later in this article, where quinine alone, even if administered for weeks continuously, will not destroy the plasmodium.

The microscope is almost indispensable for the rational treatment of many cases of malaria fevers—certainly to those who are accustomed to frequently rely upon the microscopic examination of the blood for demonstration of the malarial parasites before administering quinine even in small doses. In infected localities, however, the diagnosis of the tertian and quartan (intermittent) forms of malaria can be made practically with certainty at the bedside, from the symptoms alone, and the treatment begun at once; but in the atypical forms of those types, and in estivo-autumnal infection (remittent fever), the diagnosis is often a matter of conjecture without examination of the blood. In the severe forms of either intermittent or remittent fevers, even if the diagnosis is not absolutely certain, the quinine should be administered at once without waiting for microscopical test, because a delay in those cases may prove fatal, and we can not afford to subject our patients to the risk of such delay, even for the sake of scientific exactness.

I am aware of the fact that some of the best authorities on malaria argue that the best time to get the specific effect of quinine in the tertian, quartan, and other intermittent forms of malaria is at the close of the paroxysm, when the vitality of the protozoa is said to be the least, and just before the paroxysm, when sporulation takes place. I have tried to follow this practice, but in some way I would be mistaken as to the time when those phases in the life cycle of the hematozoa take place, and there would be a recurrence, so I adopted the plan of giving the quinine in doses of from 3 to 5 grains (0.18 to 0.3 gramme) every three or four hours, as soon as I make the diagnosis of malaria; and whether the fever is low or high, give the quinine until the fever subsides, continuing it for a few hours after the expected paroxysm—and my results have been much more satisfactory. If I am called in during the cold stage (chill), or while the fever is at its height, and the nervous symptoms—i. e., headache, backache, etc.,—are severe, I sometimes administer a sedative hypodermically, morphine 1-8 to 1-4 grain (0.008 to 0.015), with atrophine sulphate 1-150 grain (0.0004), but give the quinine at the same time. It is to be remembered that when quinine is given by the mouth it requires several hours to get its full effects, and it does not therefore add to the discomfort of the patient, as some claim, to give the quinine during the stage of pyrexia. If there is constipation I give a purgative, preferably calomel, 3 to 8 grains (0.18 to 0.5), combined with bicarbonate of sodium, podophyllin, and extract of hyoscyamus. Some physicians in this section always give an initial calomel purge before beginning the quinine, but I have not found it necessary except in cases where there is constipation, coated tongue, etc., though I believe that it is always advisable to keep the bowels and kidneys performing their eliminative functions, as the intestinal toxemia, which is coincidental with the other manifestations of the malaria explosion, unquestionably adds to the severity of the symptoms, and for that reason I usually combine with the quinine, salol 3 to 5 grains (0.18 to 0.3). I have also used with excellent success saloquinine, a new salt of quinine, which is chemically defined as the salicylic-acidester of quinine. I do not think that this latter preparation has any advantages over the combination of salol and quinine, except that it is nearly tasteless. The dose of saloquinine is from 15 to 20 grains (1.0 to 1.2) every four hours, until the antimalarial effect is obtained.

Because of the severe headache, backache, and general discomfort which accompany nearly all malarial fevers I frequently give a sedative, codeine sulphate 1-4 to 1-2 grain (0.016 to 0.03), with phenacetine, or ammonol, 2 1-2 to 4 grains (0.15 to 0.25). I desire it distinctly understood that I do not give the latter drugs for their antipyretic effect, only as analgesics.

During the stage of hyperpyrexia I have the patient sponged with cold or tepid water, and sometimes placed in a cool or even cold bath, because it makes him more comfortable. While I am not quite a believer in the theory that fever in malaria is a conservative process, an effort on the part of nature to oxidize the toxines of the disease, I am convinced that the fever, per se, in malarial diseases is never the cause of serious destructive changes, as is usually taught. As I view it, the fever is simply one of the resultant manifestations of the malarial toxemia, just as are the other symptoms, and while it is a good index, as a rule, to the degree of toxemia, it is really the latter that does the damage. I therefore do not think that we are ever justified in administering cardiac depressants with the view of "reducing fever," and while I am a great believer in the therapeutic effect of cold water in malarial and other fevers, I do not regard its antipyretic effect as being the one most desired.

The following is a favorite formula, and one with which I have cured many cases of malaria, both the intermittent and remittent forms:

℞ Quinine sulphatis, or hydrochloratis......grs. xl vel ʒj (2.5–4.0)
Salolis..................................grs. xl vel ʒj (2.5–4.0)
Codeine sulph..........................................grs. iij (0.18)
Phenacetine.............................grs. xxx vel xl (2.0–2.5)
M. Ft. in caps. No xii. S.: One capsule every three or four hours.

To insure solution of the capsule and quinine in the stomach, I sometimes followed each capsule with ten minims of dilute hydrochloric acid. In cases where I desire a quicker effect, or where large capsules can not be swallowed, I employ the Johns Hopkins Hospital method of giving the quinine in solution, though many people object very much to the taste of quinine, and physicians in general practice sometimes have to consider the desires of their patients by giving medicines in as palatable form as possible. The following formula, having the quinine in solution, is not very unpalatable, and contains the aromatic stimulant and sedative as employed by our surgeons in the Spanish-American war, and which cured many cases of the Cuban fevers where the quinine alone failed:

℞ Quiniæ sulphatis......................grs. l vel lxxx (3.12–5.0)

Acidi hydrochlor. dil..................fʒjss vel ijss (5.62–9.37)

Tr. zingiberis.........................fʒijss vel iv (9.37–15.00)

Tr. opii camph.........................fʒij vel iv (7.50–15.0)

Syr. limonis...........................................f℥ij (59–20)

Aquae, q. s. ad.........................................f℥viij (236.0)

M. S.: Tablespoonful every four hours. Take through a straw.

With children I sometimes suspend the quinine in some of the aromatic syrups, as yerba santa, in a chocolate syrup, which disguises the taste of quinine to a great extent. If the syrups are objectiorable because of a derangement of the stomach, I prescribe the bisulphate in solution, or saloquinine, which is nearly tasteless.

In the remittent (estivo-autumnal) forms of malarial fever there is usually constipation, and unless the symptoms are grave I begin the treatment with small doses of calomel, one-fourth to one grain (0.016 to 0.065) every hour until bowels have acted thoroughly. In some cases I follow this with a saline. I then begin the quinine in from three to five grains (0.18 to 0.32) every three or four hours, continuing it without regard to the height of the fever until the temperature is normal, when the intervals between doses are increased to six hours, after which the quinine is continued in tonic or prophylactic doses until there is no probability of a recurrence of the inflection.

In the comatose, congestive, or other malignant type of malaria, it is sometimes advisable to administer the quinine hypodermically. For this purpose I employ a sterilized solution of the bisulphate or the hydrochlorate, though it is rare in my practice that I see the malignant types of malaria.

Twenty-five years ago the so-called hemorrhagic malarial fever, or malarial hematuria, was one of the most frequent and fearful diseases in this country, but since then it has been gradually disappearing until it is one of the rarest diseases. So far as I can learn there have been only two or three cases in the country within the past eight years. I have had the good fortune to have seen only four cases, all of which occurred nine years ago—during my first year of practice. All four of these cases had had large doses of quinine to cure an attack of "biliousness," or to prevent an expected "chill." In one of these cases in which I was called to consult with a physician in the western part of the county, the patient's urine had very nearly cleared up, but as he had some fever I insisted upon his having more quinine. The attending physician, who had considerable experience with "yellow disease," as the natives call it, objected on the ground that the quinine would cause a recurrence of the bloody urine, but yielded to my suggestion, and the quinine was given. In six hours there was a return of the hemoglobinuria, and more severe than the first paroxysm. The physician continued to treat the case without quinine, and the patient made an uninterrupted recovery. I have had so little experience with this form of malaria—if it be of malarial origin—that I will not attempt to outline any treatment; but the four cases which I have had convinced me, not only that the quinine would do no good and is not indicated in the treatment of the disease, but that in the majority of cases, if not in all, the quinine is the exciting cause of the breaking down of the red blood-corpuscles, with the consequent hemoglobinuria.

From the foregoing remarks it is clear that I consider quinine the drug par excellence in the treatment of nearly all forms of malaria, but unfortunately I have to treat many people who have a marked idiosyncrasy for that agent. I have seen persons in whom even small doses of quinine would produce great discomfort and even alarming symptoms, such as severe tinnitus aurium, nausea and vomiting, urticaria, or even marked depression; in others small doses can be tolerated, but not in sufficient quantities to destroy the plasmodium. In such cases, where these unpleasant effects can not be prevented by the use of the bromides, etc., we are compelled to use the more unsatisfactory and less certain substitutes for quinine. Of these I get the best results from methylene blue in two grain doses (0.13) every three or four hours. I usually give it in capsule with powdered nutmeg and small does of codeine, which seems to prevent the strangury and nausea which sometimes follow the use of the methylene blue when given alone. I have used methylene blue in a large number of cases of malaria, and I know that it has marked antiperiodic powers, though it is not as certain in its effects as quinine. In those case where the "untoward effects" of quinine are not so marked, but sufficiently so to prevent its use in large enough doses to obtain its full antimalarial effect, I combine the methylene blue, grain i (0.065), with quinine sulphate, grains ij (0.13), with excellent results. In some cases, where the sulphate or hydrochlorate is not tolerated, the other alkaloids of cinchona are well borne. I have seen a number of persons who were always very unpleasantly affected by quinine, but who could take some of the proprietary

"chill tonics," which are said to contain amorphous quinine, in such doses as to get their antiperiodic effect. Salicin has some reputation as a substitute for quinine, and I have used it in a few cases, but to get specific effect the doses have to be so large, fifteen to thirty grains (1.0 to 2.0), that in some cases it is not well tolerated by the stomach. Phenocoll hydrochloride, in daily doses of from twenty to thirty grains (1.5 to 2.0), is said to be another excellent substitute for quinine in the intermittent forms of malaria.

One who has not made frequent microscopic examinations of the blood in malarial diseases can hardly realize the marked and even grave changes that take place in the blood in a very short time. The blood count and hemoglobin test do not tell half the tale of the destruction of the red blood-cells—indeed, it is almost unnecessary to make these tests, as a glance at the irregular sizes and shapes of the erythrocytes (poikilocytes), as well as the fragments of the red blood-cells, is sufficient indication to the busy practitioner that iron and arsenic are demanded.

In some cases, particularly the chronic forms of remittent (estivo-autumnal) fevers, with enlarged spleen, etc., the quinine does not seem to have its specific effect until the anemia has been partially overcome. The following case, briefly related from memory, illustrates this point, as well as the fact that all continued fevers that do not respond to the use of quinine in a few days are not typhoid:

Girl, aged fourteen. Was brought from a neighboring county to me for treatment on June 21. History of having had fever for three or four weeks. Temperature 102° F., pulse 120, very anemic, enlarged spleen, etc. Microscopic examination of a fresh blood smear showed large numbers of hyaline, crescentic, and extracorpuscular forms of the estivo-autumnal parasite. Apparently no leukocytosis, but the white blood-cells were heavily pigmented. Marked poikilocytosis, with many fragments of red blood-cells, and other evidences of a severe hemocytolysis. Because of lack of time no blood count or hemoglobin test was made. Patient placed on full doses of quinine, iron and arsenic. Fever continued, but general condition of patient improved. Quinine discontinued on fifth day, but iron and arsenic were continued.

On the seventh day another examination of the blood was made, which showed numbers of the parasites present, but there were less evidences of anemia. She was then given quinine in solution, 4 grains (0.25) every three or four hours for five days, and two days after the quinine was left off another examination of the blood was made, which demonstrated the presence of a few parasites, but the blood-corpuscles were more nearly normal. She was so much improved that she was carried to her home, but the quinine was continued another week, and the arsenic and iron, with quinine in tonic and prophylactic doses, were continued for another month. She made a slow but certain recovery, and a few weeks ago I learned that she was in fine health.

For the treatment of the anemia following both the intermittent and remittent forms of malaria, the so-called "malarial cachexia," the following formula has given me excellent results:

℞ Quininae sulph....................ʒjss (6.0)
Ferri redacti....................ʒj (3.9)
Ext. nucis vomicae....................grs. viij (0.52)
Acidi arseniosi....................gr. j (0.065)
Ext. colocynth. com....................grs x vel xv (0.65-0.97)
Podophyllini....................grs. j vel iij (0.065-0.19)
Mellis, q. s.
M. Ft. in pilulæ No. lx. S.: One pill after each meal.

Many people have the idea that negroes are but slightly susceptible to malaria, but I am convinced, for many reasons, at this time, that they are even more susceptible to malarial fevers than are the whites, and I think I can give statistics showing that I have excellent grounds for such an opinion. Of course malaria is essentially the same disease in the negro as in the Caucasian, but there are certain differences in its manifestations in the two races, that variations in treatment are often necessary. I had intended to discuss these differences in this article, but I fear that I have already taken up more than the space allotted to me; but in the near future I will prepare a paper entitled "Malaria in the Negro," in which I will go into detail on that great neglected subject.—*Alabama Medical Journal.*

### DYSMENORRHEA AND ALLIED MANIFESTATION.*

In my choice of a theme for presentation this evening, I have been influenced not only by the suggestions of your esteemed chairman, but also by my estimate of the importance of the subject to general practitioner and specialist alike. It is probable that physicians are consulted more frequently for dysmenorrhea than for any other one gynecologic disturbance. In view of this fact and the consequent abundant opportunities for the study of this affection clinically, our lack of exact and definite knowledge concerning it is remarkble. Unfortunately, I can not hope to add much to what is already known; but believing that it is distinctly helpful to set down in order from time to time such conclusions as we may have drawn, I venture to present a brief resumé from the point of view of professional experience, starting where the physician commonly begins in the consideration of the case, namely, with dysmenorrhea as a symptom.

We are all well aware of the tendency on the part of many practitioners to regard as trivial a degree of menstrual suffering which, if associated with the performance of such other physiologic functions as digestion or elimination, would receive assiduous attention. In order that we may not be accused of going to the other extreme, and exaggerating the importance of insignificant sensations, it may be well to state what may be legitimately included in the term menstrual molimena.

It is the exceptional woman who is not conscious of some variation in bodily states immediately preceding or during the menstrual period. This may be limited to an exacerbation of her normal energy, an impulsion and ability to do a greater amount of work than usual, before the appearance of the flow. Or quite the opposite may be noted, and disinclination and lassitude may take the place of increased nervous unrest and activity. Vasomotor disturbance may be shown by greater susceptibility to cold, some superficial capillary contraction, or by the sudden flushing of capillary dilation, and a sense of heat throughout the body, the whole

* Read by invitation by Ella B. Everitt, M. D., Professor of Gynecology, Woman's Medical College of Pennsylvania, Philadelphia, before the section on Obstetrics and Gynecology of the Wayne County Medical Society, December 21, 1903.

resembling the heat flash of the menopause, but in much milder form and without the associated dyspnea. In women who are not without sexual feeling, there is commonly an increase in the excitability before, during or immediately after the period.

Passing beyond these general sensations, there may be local discomfort, as a sense of weight or fulness in the pelvis, or an almost indefinable sense of pelvic tension which might be regarded as a menstrual aura in some instances. Soreness and swelling of the breasts increasing throughout the week preceding the flow are invariable forerunners in some patients. Diarrhea is a frequent accompaniment of the first day. Even a slight aching in the pelvic, sacral and lower abdominal regions, and aching fulness in the external genitalia should not be regarded as abnormal. But from this point onward, we pass to the realm of the pathologic, and reach distinct dysmenorrhea, varying from simple cramps to agonizing suffering.

CLASSIFICATION.

Nothing is more important in our consideration of this really complex subject than a practical classification of the forms of dysmenorrhea based on its etiology. A generally accepted and rational one is as follows, the order being that of relative frequency:

1. *Neuralgic.* Under this head are included those cases unassociated with demonstrable structural abnormality in the pelvic organs, or with local disease. This form presupposes an underlying condition of lowered nervous vitality, which may be chronic or temporary. It is the common manifestation of the neurotic temperament; or, of nervous exhaustion, the result of overwork or excessive mental strain, in patients usually strong nervously. It may be due also to such constitutional conditions as anemia, chlorosis, gout, rheumatism, malaria, or syphilis, wherein the blood is so altered as to profoundly affect the nutrition of the nervous system. It has been objected that "for pelvic pain at the menstrual time, bred by starving or irritated nerves in some remote part of the nervous system, the term dysmenorrhea is inappropriate, for it does not appear that the pain is due to menstruations," * and the

*Reed: Text-book of Gynecology.

word menorrhalgia has been suggested (by Massey) as a substitute. It seems probable, however, that by the menstrual functioning there is just enough additional strain on the already weakened nerve tone to cause a local, pelvic expression, hence we may regard menstruation as bearing at least a partial causal relation to the pain in the neuralgic form, and we may retain the term as convenient and readily understood.

2. *Congestive.* When the pelvic organs are effected by inflammatory disease, or are the seat of tumors favoring abnormal vascularity of the parts, the physiologic congestion of the menstrual period augments the pain of the intermenstrual period, hence the term congestive dysmenorrhea. This is the so-called inflammatory form of some authors.

3. *Obstructive.* By this is meant those cases due to mechanical interference with the escape of fluid from the uterine cavity; the obstruction itself being due to stenosis of the cervical canal, from sharp flexions, inflammatory adhesions, congenitally small orifice or the presence of polypi. Some authorities tend to include under this class those cases in which from defective development or abnormal histologic conditions in the uterine wall, the blood breaks through *into the cavity* with difficulty. While it is important in dealing with cases of dysmenorrhea to remember this possible etiology, this is not the usual understanding of the subdivision.

Whether or not the retention of the menstrual fluid within the cavity is really caused by flexions, has been a much discussed question. By some observers, it is claimed that even in flexed uteri, the orifices are open during the flow, and that they do not interfere with the discharge. By others it is stated that in cases of flexion an abnormally sensitive condition of the mucous membrane exists at the internal os, which when the menstrual blood makes pressure upon it is responsible for the pain. It is not within the scope of this paper to argue this point. Suffice it to say that while many patients with marked flexions menstruate painlessly, as a rule there is, with this condition, a resulting endometritis to which the pain at the menstrual period may be directly attributed, and the curing of which relieves the dysmenorrhea even though the flexion is only partially corrected, or subsequently returns.

4. *Ovarian.* Ovarian dysmenorrhea is merely an exaggeration of the intermenstrual pain by the congestion of the physiologic period, in patients suffering from ovarian disease.

5. *Membranous.* The normal intermenstrual growth of the uterine mucous membrane is a "preparation for a possible future embryo; the tissue of the decidua menstrualis is the forerunner of the decidua graviditatis; if an ovum wherever it is discharged, be fertilized, it attaches itself to the thickened uterine wall, the tissues become the decidua graviditatis, pregnancy follows, and the decidua is not discharged until the time of parturition. If, however, fertilization does not take place, there is no attachment, the tissues degenerate and become the decidua menstrualis,"* to be discharged insensibly with the next flow. But if, as the result of chronic endometritis, the membrane in its formation shows marked hypertrophy and excess of fibrous tissue, instead of disintegrating, it maintains its membranous character and is exfoliated in sections or as a complete cast of the entire uterine cavity. The pain is due to the expulsive effort of the uterus as it endeavors to separate and cast off the membrane.

### DIAGNOSIS OF VARIOUS FORMS.

The characteristics of the pain in the different forms of dysmenorrhea are sufficiently distinct and pronounced as to *suggest,* if not definitely indicate, the casual factors. A careful study of the pain with relation to its location, time of onset, duration, character, severity and recurrence, will amply repay the physician in every case.

In the neuralgic form, we note all grades of severity from slight cramp to violent nerve explosions. The location is commonly uterine (hypogastric), but may be ovarian. Its onset is usually simultaneous with the appearance of the flow, increasing in severity from one to several hours, and subsiding gradually after a free discharge has been established, or ceasing suddenly after an attack of vomiting or faintness has produced general relaxation. The pain is undulatory, or a series of pains, resembling those of uterine contractions in labor. Each pain gradually shades on to a maximum intensity, remains a few seconds at this point, then shades

*American Textbook of Physiology.

off to partial or entire freedom from discomfort. After a short interval, another pain of the same character but more severe, follows, until the height of the nerve storm is reached, when it subsides in one of the two ways above indicated. The association of neuralgic pains in other parts of the body, the absence of pelvic lesions, and the fact that the function is painlessly performed when the subject is under no nervous or physical strain, confirm the diagnosis. Teachers and students who suffer from this form not infrequently menstrate in perfectly normal manner during vacations.

The pain in the congestive type is more or less constant. It is especially noteworthy that it is *acquired*, after some years of painless menstruation. The location is usually median, low in the abdomen, or in the back over the sacrum, although in tubo-ovarian disease it may be inguinal, spreading throughout the pelvis and down the thighs. It is more apt to be a dull or severe *aching* than acute pain, and is relieved as a rule after the flow has lessened the congestion.

In the obstructive and membranous varieties, pain is intermittent and spasmodic, and may precede the flow several hours. It is usually hypogastric and may be very severe. In the membranous form, it is often of the character of a partus miniaturus, leaving the patient prostrated after the expulsion of the membrane. If the latter is shed in sections, there may be a series of pains, each series culminating when the fragment has passed the cervical canal. The expulsion of clots often marks the cessation of pain in the mechanical type. In all these four forms, the recti muscles are involuntarily held more or less rigid while the patient is suffering.

Ovarian dysmenorrhea is an *acute* pain in the ovarian region, or *an aching* in the uterus, together with the ovarian pain. Gastric reflexes are common. The pathologic condition of uterine appendages will serve to distinguish it from the neuralgic form.

*Intermenstrual Pain.*—In addition to these regular forms of menstrual suffering, two other manifestations are especially important, viz.: menstrual headache, and what is erroneously called "intermenstrual dysmenorrhea," better, the "mittelschmerz" of the Germans. By the latter is meant an attack of pain usually inguinal, occurring with greater or less regularity, about midway between the menstrual epochs, sometimes a little earlier or a little later. This phenomenon is almost invariably seen in neurasthenic subjects, and develops in the later menstrual life. The pain may come on, on two or three successive days—a cramping paroxysm lasting one or two hours and then subsiding; or it may continue longer, with no recurrence. It may alternate, returning only every other month, or it may have the same periodicity as the flow itself. When located in the right side, it is not infrequently mistaken for appendicitis or appendicial colic, and the appendix is removed. It is needless to say that the pain is unaffected by this surgical procedure. I have under my care at present a patient who has been suffering in this way for seven years, during which time she has had the operations of curettage, appendectomy, and even the removal of the hemorrhoids, all undertaken to relieve the intermenstrual pain which she still has. The affection is especially obstinate, resisting almost all manner of treatment. Two theories as to the cause have been advanced: 1st. That the pain is due to the tension of ripening follicles, a theory which is hardly tenable, as follicles are ripening constantly throughout the entire interval between periods. 2d. The second theory, that at the middle of the intermenstrual period the recession of blood pressure in the pelvis reaches its ebb, offers a more probable explanation. (This is quite independent of the rise in general blood pressure, as shown by the so-called "Stevenson's wave.")

It will be remembered that the menstrual cycle as given by Leopold shows:

| | |
|---|---|
| Tumefaction of the mucosa | 5 days. |
| Menstrual flow | 4 days. |
| Regeneration of mucosa | 7 days. |
| Resting of mucosa | 12 days. |
| | 28 days. |

From my own observations of patients having mittelschmerz, I should be inclined to believe in a nervous recession corresponding to the vascular one, and to consider this pain a nerve cry similar in character to some neuralgic dysmenorrheas.

*Menstrual Headache.*—The headache related to the menstrual period is one of the most persistent and trying forms of this malady. The head pain is sometimes prodromal, coming on one, two or even three days before the flow appears, and gradually subsiding a few hours after it is established. Again, it may accompany or follow the flow, but in the same individual it usually recurs at a similar time with each period. The headache may be vertical, frontal, or less frequently occipital. Sometimes it is a distinct hemicrania, often of a neuralgic character. Occasionally neuralgic headache will alternate with menstrual cramps of neuralgic origin, one or the other being the accompaniment of each period, but both not occurring in the same month. The attack may begin as a dull, confused sensation throughout the head, gradually centering as pain on one or the other side. Or, it may be distinctly neuralgic from the first, affecting the superficial nerves on one entire side of the head. In some instances, throbbing of the vessels indicates the congestive character, while in others the vascular tension is decidedly low. Disinclination for food, or nausea and vomiting may be present.

The etiology of these headaches is probably different in different forms. The neuralgic type seems closely related to mittelschmerz, in that it appears to be the result of lowered nervous tone. Whether or not there is a nervous center presiding over the function of menstruation, the nervous system is unquestionably profoundly affected by the menstrual period. In some cases this depression is manifested in the abdominal plexuses; in others, in superficial nerves in remote parts of the body; in others, in the central system.

### THE HYGIENE OF THE MENSTRUAL PERIOD.

Before even mentioning the treatment of these various conditions, the question of the hygiene of the menstrual period may be pertinently introduced. Two objects are to be attained in the management of a menstruating patient: One, the maintenance of the best possible general health, and the other, the equalization of the circulation throughout the body. While the calling of two much attention to the pelvic organs and their activity is unwise and undesirable, a certain thoughtfulness for hygienic conditions should be encouraged in all cases. Avoidance of extra nervous or mental strain is important, and to this end, the school surroundings of young girls should be looked into. The analyses of thousands of cases of dysmenorrhea in schoolgirls shows an increase in pain with the pursuit of studies, and usually there is at least a lurking conclusion that the mental effort is responsible. In reality, well-regulated mental and physical work is beneficial : to the *lack of adequate physical care and training* during the school life, the results in ill health should be attributed. It is essential, however, that physiologic periods should be regarded, hence the desirability of separate schools for young girls, and such an elastic educational system as will call for no unusual mental effort during the period. The interruption of ordinary routine is seldom necessary or advisable. Exercise in moderation and in the open air during the period promotes painless menstruation. Under conditions of average health, there need be no interruption of the usual bathing habits. It must be remembered, however, that where there is an *acquired* habit of abstaining from the bath during the period, this practice can not suddenly be broken up, but the daily bath must be gradually resumed. Warm, light clothing, with ample protection to the ankles, is essential to an equable distribution of blood in the body, and the prevention of pelvic congestions. The vaginal douche after the flow is unnecessary for personal cleanliness, and, especially in unmarried women, should be discountenanced.

### TREATMENT OF DYSMENORRHEA.

In regard to treatment, it is not my purpose to enter into a detailed recital of what may be done to cure dysmenorrhea. The means for the relief of the paroxysm are well known. Certain definite indications, based on the casual factors, must be met, and the methods of doing this are more or less uniform throughout this country. Systemic states must be appropriately combated; flexions and misplacements must be corrected, cervical stenoses overcome; curable inflammatory diseases systematically treated, and hopelessly diseased appendages removed. But *the method of approach,* the manner of investigating the case, calls for more than a passing word. We are all well aware of the too frequent haphazard treatment of these patients; with no examination and little questioning, an antispasmodic or anodyne (well if it is not an opiate or alcohol in some form) is prescribed, and the patient is given the general direction, to "take douches." No definite instructions are given as to how or when this should be done, or how the necessary apparatus should be cared for, and there is no clear idea on the part of the doctor as to what is to be accomplished within the pelvis by this procedure. I do not know how to condemn sufficiently strongly this kind of practice, and while I would not be understood as advocating the indiscriminate examining of women, married or single, it is either a false regard for the feelings of the patient, or gross negligence which allows treatment so utterly at random. Systematic local treatment aseptically practiced *when indicated* undoubtedly has great value. But unless administered intelligently with a clear idea of existing pelvic conditions, and of the diseased states amenable to such treatment, it is worse than useless, is bound to bring the method into disrepute, and is often responsible for grave infective lesions calling for subsequent sacrificial surgery.

The importance of attaching a correct valuation to the pelvic lesion discovered by examination can not be too strongly emphasized. We dare not jump at conclusions. That an anteflexion exists is not final proof that it is the cause of dysmenorrhea. This may be due to a prolapsed ovary quite overlooked, or to an exhausted nervous system calling for rest, and *not* for any local medication or tinkering. On the other hand, a markedly anemic condition does not preclude the existence of serious pelvic deformities calling for surgical interference before normal functioning can be performed. Let me therefore close with the strongest possible plea for the study of each patient as an individual, and not merely as a case of stenosis, or of tumor or of endometritis. Only by such conscientious and painstaking investigation can we discharge our duty to our patients and our profession.—*Detroit Medical Journal.*

